# Consumer Perspectives on Trust in and Benefits of Artificial Intelligence in Health Care

**DOI:** 10.1001/jamanetworkopen.2026.26916

**Published:** 2026-08-04

**Authors:** Tuan Duong, Stefanie Plage, Leanna Woods, Miriam Dillon, Quita Olsen, Titus Kirwa, Anna Brown, Wenyong Wang, Clair Sullivan

**Affiliations:** 1 Faculty of Health, Medicine, and Behavioural Sciences, Queensland Digital Health Centre, The University of Queensland, Brisbane, Queensland, Australia; 2 Family Medicine Department, Hue University of Medicine and Pharmacy, Hue University, Hue, Vietnam; 3 Faculty of Humanities, Arts, and Social Sciences, School of Social Science, The University of Queensland, Brisbane, Australia; 4 Centre for the Business and Economics of Health, The University of Queensland, Brisbane, Queensland, Australia; 5 Toi Āria: Design for Public Good, College of Creative Arts, Massey University, Wellington, New Zealand; 6 Metro South Hospital and Health Service, Brisbane, Australia; 7 Metro North Hospital and Health Service, Brisbane, Australia

## Abstract

**Importance:**

Artificial intelligence (AI) has the potential to improve patient-centered care, yet several AI health care projects have failed due to public backlash, underscoring the importance of social license (informal public acceptance of AI in health care, grounded in trust and expectation of public benefit). How consumers conceptualize social license remains largely unknown.

**Objectives:**

To explore social license for AI in health care among consumers and to identify strategies for achieving it.

**Design, Setting, and Participants:**

A participatory qualitative study using the comfort board method was conducted across workshops in Queensland, Australia, in September 2025, applying abductive reflexive thematic analysis. Participants were recruited using a mix of convenience and purposive sampling. Eligibility criteria included being aged 18 years or older, residing in Queensland, self-reported receiving care in a Queensland health care facility within the past 24 months, and able to communicate in English.

**Main Outcomes and Measures:**

Concept of social license for AI in health care and strategies for achieving it.

**Results:**

Thirty-four participants (21 [62%] female) were included, with diverse ages (16 [53%] aged 31-60 years), countries of birth, disability statuses, and AI familiarity. Key themes of social license for AI included relational engagement, structural support, and performance reliability. Performance reliability was critical in short-term care, whereas relational engagement and structural support were more salient in long-term care. According to participants, achieving social license would require AI systems to function as supportive clinical tools, governed by strict stakeholder-driven standards and developed through codesign that aligns them with care needs, contexts, and patient-clinician interactions.

**Conclusions and Relevance:**

This qualitative study of consumer perspectives on AI in health care found that social license for AI is a conditional and dynamic construct not a fixed state; structural, performance, and relational factors intersected to shape social license. The findings provide evidence-based recommendations for stakeholders designing and implementing AI in clinical settings, highlighting the need for AI tools designed to support both consumers and clinicians in delivering care that is personalized, empathetic, and responsive to patient needs.

## Key Points

**Question:**

How can informal public acceptance of artificial intelligence in health care be achieved?

**Findings:**

In this qualitative study involving 34 participants, social license emerged as a conditional construct shaped by relational engagement, structural support, and performance reliability. Performance reliability was prioritized in short-term care, whereas relational engagement and structural support were more prominent in long-term care.

**Meaning:**

These findings suggest that social license for artificial intelligence is a conditional, dynamic construct, and people accept artificial intelligence when it helps deliver care that is personalized, empathetic, and responsive to patient needs.

## Teaser

This qualitative study explores social acceptance of artificial intelligence in health care.

## Introduction

Artificial intelligence (AI), computational systems that can perform tasks that are commonly performed by human intelligence,^1^ has emerged as a transformative technology, demonstrating potential to enhance clinical practice and patient outcomes.^2,3^ Despite the increasing need for AI-enabled care, many nations do not yet have a fully unified national framework guiding the use of AI in health care.^4,5^ International legal frameworks, such as the European Union Artificial Intelligence Act and the US National Policy Artificial Intelligence Framework, provide additional contexts for evolving international standards.^6,7^ Beyond legislation, AI development and deployment must adhere to ethical standards. The World Health Organization provides key principles for ethical AI use in health care, including autonomy, well-being, transparency, accountability, inclusiveness, equity, and sustainability.^1^ However, successful implementation of AI in health care requires more than compliance with laws and ethical approval. Several health data projects have met both legislative and ethical standards and yet failed due to public backlash.^8,9,10^ This highlights the importance of social license as a critical factor for the success of AI in health care.^10,11,12^

Social license refers to a dynamic, informal, and tacit form of public acceptance granted for organizations undertaking AI-related activities in health care contexts, such as developing, deploying, or governing these technologies.^12,13,14,15,16,17^ Importantly, social license is not equivalent to legal compliance or regulatory approval; rather, it is grounded in public trust and expectation that these activities will align and promote public benefits.^12^ While perceptions about benefits can potentially affect trust, trust in turn moderates how these perceptions influence public responses to AI.^12,14^ Together, trust and perceptions of benefits are central determinants of the social license for AI in health care.

Social license for AI health care includes both the sharing of health data for AI development and the use of AI technologies in clinical practice. Previous literature indicates increasing consumer awareness of health data sharing in the context of AI, with generally positive attitudes toward sharing personal health data for AI purposes.^18,19,20,21,22^ Emerging evidence also suggests broad support for AI as an assistive tool in clinical decision-making, with many consumers expressing trust in AI-enabled diagnosis and treatment.^19,20,23^ However, these studies focus mainly on consumer perspectives toward specific AI-related activities, particularly data sharing, leaving a notable gap in understanding how consumers conceptualize social license for AI in health care. Addressing this gap requires examining the foundations of social license from health consumer perspectives, with particular attention to trust and perceived benefit. Our study focused on the overarching research question: How can a consumer social license for AI in health care be achieved? Specifically, our study aimed to explore social license for AI in health care among consumers and to identify and propose strategies for achieving social license for AI in health care.

## Methods

### Study Design

We used a participatory qualitative design grounded in an interpretivist paradigm, using scenario-based, interactive workshops based on the comfort board method.^24^ In this approach, social license for AI in health care was explored through its key determinants: perceived trust and benefit. These determinants also form the 2 axes that underpin the comfort board’s diagonal comfort scale (eFigure 1 in Supplement 1). In our study, comfort served as a practical approximation of social license. We followed the Standards for Quality Improvement Reporting Excellence (SQUIRE) reporting guideline when developing, conducting, and reporting the study^25^ (eMethods 1 in Supplement 1).

### Reflexivity

The senior author (T.D.) is a PhD student with a background as a general practitioner with experience in primary care and medical education, bringing a patient-centered orientation to the study. The coauthors (S.P., L.W., M.D., Q.O., T.K., A.B., W.W., and C.S.) contributed diverse expertise across public health, digital health, clinical practice, sociology, and participatory design, which informed both the design and interpretation of the study. We considered the possibility of prior relationships between the research team and participants, but none existed. Ethical approval was granted by The University of Queensland Human Research Ethics Lower Risk Panel.

### Participants and Recruitment

The study was conducted in Queensland, Australia, a culturally diverse state.^26^ Participants were recruited using a mix of convenience and purposive sampling. Eligibility criteria included being 18 years or older, residing in Queensland, self-reported having received care in a Queensland health care facility within the past 24 months, and being able to communicate in English. Recruitment materials were distributed through consumer and advocacy networks and diverse public communities.

Our sample size was guided by the concept of information power.^27^ Our study had a focused aim, included relevant participants, and generated rich scenario-based discussions, indicating that fewer participants were sufficient to generate a sufficiently rich dataset.^27^ During analysis, later small group discussions added nuances but did not introduce new themes, indicating that the dataset held sufficient information power. Accessibility needs were identified and addressed during recruitment and workshops, including travel assistance, tailored seating, and Braille boards for participants with visual impairments. Written informed consent was obtained from all participants before their participation in the study. The informed consent included permission to record and publish participant quotations.

### Scenario Development

A multidisciplinary team developed 2 realistic scenarios that differed in transparency, data use, and organizational involvement to prompt diverse responses. Whereas scenario 1a and 1b focused on long-term care settings, scenarios 2a and 2b addressed short-term care settings (eMethods 2 in Supplement 1).

### Data Collection

Two in-person interactive workshops were conducted in September 2025 at the university campus, following a structured guide developed by the research team (eMethods 3 in Supplement 1). Participants first completed a brief survey capturing demographics and AI literacy (eMethods 4 in Supplement 1). They were then introduced to the concept of comfort and the comfort board, a grid mat with trust and benefit axes ranging from 0 to 7, with 0 indicating no trust or perceived benefit and 7 indicating maximum trust or perceived benefit. In this context, benefit and trust referred to participants’ perceived level of benefit and trust in the decision or action in each scenario when imagining themselves as the main character. For process familiarization, participants began with scenarios 1a and 1b, positioning themselves on the matrix to show their initial comfort and briefly explaining their choices.

They then joined small group discussions of up to 7 with 1 facilitator. The discussions began again with scenario 1a, followed by 1b, 2a, and 2b. Using tokens on tabletop comfort boards, they reflected on the positions for each scenario and discussed what might increase their comfort. After completing all 4 scenarios, participants engaged in a discussion on how comfort with AI in health care can be increased, organizing their insights on a thematic map (eFigure 2 in Supplement 1).

### Data Analysis

All quantitative data and analyses were used in a supportive capacity to contextualize qualitative findings. Aggregated comfort board positions provided a visual summary of participants’ comfort levels and their shifts across scenarios. Descriptive statistics were used to summarize positions distributions and changes in position means. The Wilcoxon signed-rank test was used in an exploratory manner to examine the trust and benefit changes within scenarios (with a 2-sided *P* < .05 indicating statistical significance).

Group discussions were audio-recorded and transcribed verbatim, with support from the web-based application Otter.ai (Otter.ai Inc). Transcripts were analyzed using abductive reflexive thematic analysis approach in NVivo, version 14 (Lumivero), guided by a conceptual breakdown of social license, including trust and perceived benefit, with additional constructs generated inductively from the transcripts. T.D., S.P., and M.D. independently coded transcripts and met regularly to refine the coding framework, followed by broader team meetings to finalize themes. Participant-generated thematic maps were used to support theme refinement. An audit trail documented key decisions throughout the study.

Insights from the thematic analysis were synthesized into evidence-based recommendations for achieving social license and mapped to relevant actors across 3 conceptual horizons of digital health transformation framework (eFigure 3 in Supplement 1).^28^ Details of data analysis are illustrated in eMethods 5 in Supplement 1.

## Results

A total of 34 participants (21 [62%] female and 13 [38%] male; 16 [53%] aged 31-60 years) were recruited across 2 workshops (18 in workshop 1 and 16 in workshop 2). Of 27 participants with available information on country of birth, 10 (37%) were from Australia, 9 (33%) from East or Southeast Asia, 6 (22%) from Europe, and 2 (8%) from Fiji and Zambia. Of 28 participants with available data, 9 (32%) reported having disabilities. Of 30 participants with available data, 16 (53%) reported being moderately familiar with AI (Table 1).

**Table 1.** Participant Characteristics

Characteristic	No. (%) of participants (N = 34)
Gender	
Male	13 (38)
Female	21 (62)
Age range, y (n = 30)^a^	
18-30	5 (17)
31-60	16 (53)
>60	9 (30)
Country of birth (n = 27)^a^	
Australia	10 (37)
East or Southeast Asia	9 (33)
Europe	6 (22)
Fiji or Zambia	2 (8)
Disability status (n = 28)^a^	
Yes	9 (32)
No	19 (68)
AI in health care familiarity (n = 30)^a^	
Not familiar	1 (3)
Slightly familiar	8 (27)
Moderately familiar	16 (53)
Very familiar	5 (17)

Abbreviation: AI, artificial intelligence.

^a^ Sample sizes varying because some participants did not answer all questions.

Comfort levels across scenarios are summarized in Table 2. Average trust and perceived benefit levels remained above 4 on a 0- to 7-point scale, with benefit consistently rated higher than trust. From scenario 1a to 1b, trust and perceived benefit declined, supported by a statistically significant decrease in trust levels (W = 219; *r* = 0.23; *P* = .01). In contrast, from scenario 2a to 2b, trust and perceived benefit increased, with benefit showing a statistically significant increase (W = 47; *r* = 0.73; *P* = .01). The Figure visualizes comfort levels of participants at each scenario and the movement of selected positions in scenario 1 and scenario 2.

**Table 2.** Comfort Levels Across the Scenarios

Scenario	Mean (SD) comfort level	
	Trust	Benefit
1a	5.1 (1.6)^a^	5.3 (1.5)
1b	4.4 (1.8)^a^	5.1 (1.6)
2a	4.5 (2.0)	5.2 (1.6)^a^
2b	4.8 (1.7)	6.0 (1.3)^a^

^a^ Significant difference between trust or benefit when scenario 1 or scenario 2 changed.

**Figure.**
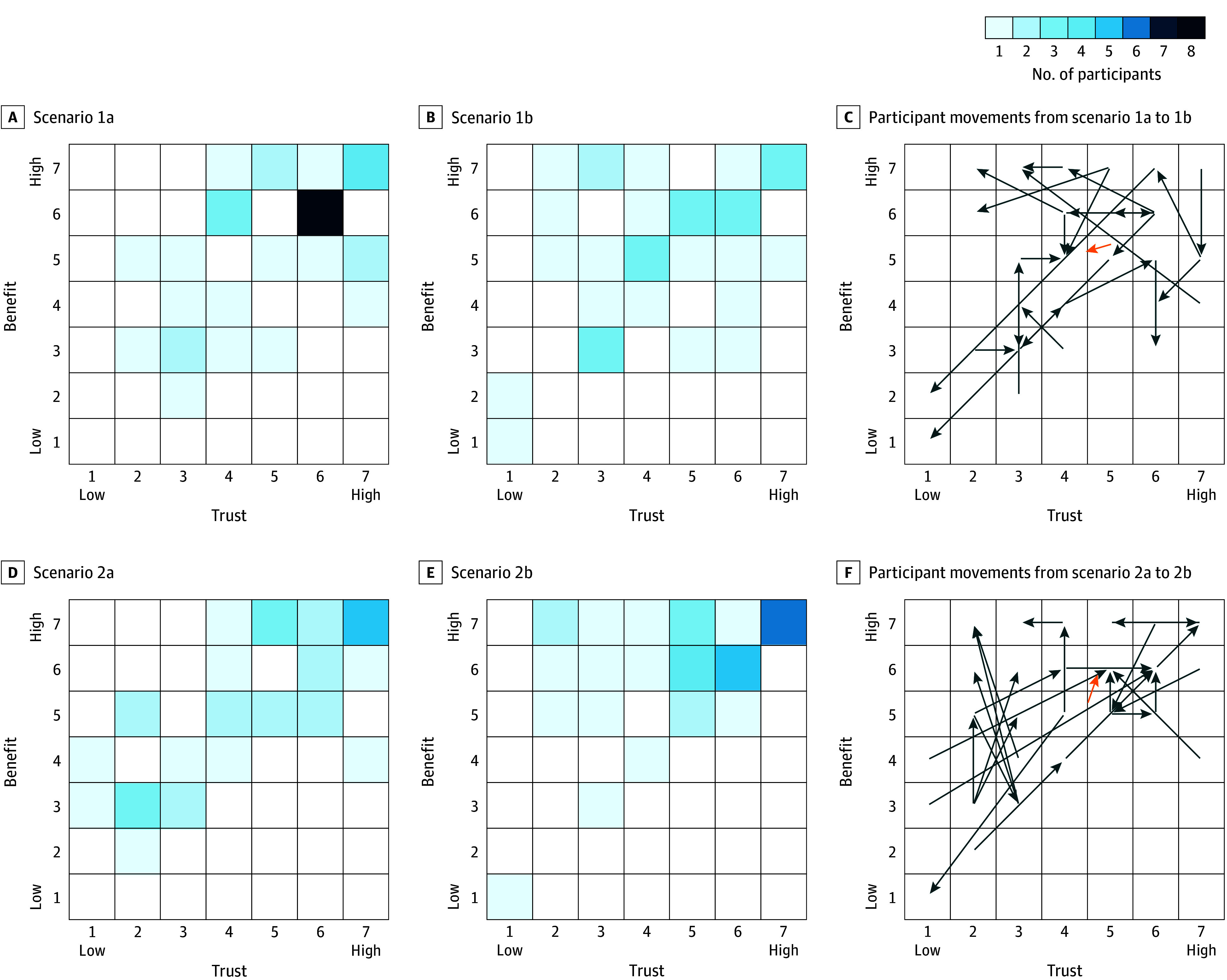
Grid Graphs of Participant Positions on the Comfort Board Scenario 1a and 1b focused on long-term care settings and scenarios 2a and 2b on short-term care settings. Darker colors indicate a higher number of participants selecting that position. Narrow arrows indicate movement of the participants; orange arrows, overall movement position of all participants.

We identified 3 primary themes of social license for AI in health care: relational engagement, structural support, and performance reliability. Subthemes of each theme are explained in Table 3. Evidence -based recommendations for achieving social license are highlighted in Table 4.

**Table 3.** Themes and Quotations of Social License for AI in Health Care

Theme	Subtheme	Quotations (participant identifier)
**Relational engagement**		
Interpersonal factors that shape health consumer comfort with AI	Trust in clinician: high trust in clinicians	“I’ve trust in my doctor a certain degree, and so far, everything he’s been doing been working well.” (Participant 35) “I’ll be the position of grace…High respect professions. I will not be too worried. There’re the people actually saved my life. They know what they’re doing…” (Participant 63)
	Patient-clinician interaction: patient-clinician interaction is critical and irreplaceable	“I have a relationship with my doctor. Because they’ve got a doctorate, they’re very well experienced.” (Participant 24) “Instead of going to see a GP, you’re going to see a computer, she’s going to sit there and diagnose you and choose what to do and prescribe things for you. We’re going to end up completely removing…the person, the human practitioner…it’s frightening.” (Participant 45)
	Relationship continuity: value of relationship with health staff when introducing AI	“…If the doctor gives me like the diagnosis based on like these parameters [of AI], I would just listen to it.” (Participant 66) “If I knew somebody who I trusted, who was a health professional who assured me that it was a genuine process, there was some fluffy bits, but it was genuine and there was good work being done on it, then I might trust it more.” (Participant 34)
	Shared decision-making: shared decision-making about using data for AI and AI integration	“I don’t want to find out that all my personal data has been collected [for AI] without me having agreed…” (Participant 30)
**Structural support**		
Organizational and infrastructural factors of health systems that shape the integration of AI	Data governance: strong regulatory frameworks and legislations	“Maybe the hospital is about to use your data for AI…You’ll say yes, yes, yes, because it’s not your concern. I just want to feel better again.” (Participant 35) “Your life is more vital, data will be a secondary thought, of course.” (Participant 63) “…We should have the legislation actually to identify where responsibility lies.” (Participant 44) “I would like to know some principle they use the data,…how do they gathering the data? How do they store it, and what’s the principle they gathering the data?” (Participant 96)
	Privacy and security: protection of health data and privacy of consumers	“Even if it [data] was de-identified, I’m not sure about that…I’m a bit cynical about that.” (Participant 34) “It [AI] is not acceptable to use is when it starts harming you…For example, don’t scam me.” “It’s private companies. I don’t know about their ethics. I don’t know about how they make profit.” (Participant 96)
	Accountability: ensuring clear responsibility for decisions and outcomes	“…We’re always on about how researchers or clinicians need to be trained in how to communicate with consumers, and how consumers have rights to information and to proper being properly informed at a level which satisfies both the clinician and consumers. And there isn’t enough of it yet.” (Participant 34) “…If it [AI] does give you information that’s life and death and it results in an adverse outcome, then who do we hold responsible? And how do we change that, if there’s no one accountable, we can’t do that. There has to be someone that looks at what’s going on, understand what’s going on.” (Participant 50)
	Transparency: transparency of health data use and AI systems	“This one is totally unacceptable, because the patient actually finds it out by accidentally, unacceptable respect…You use patients to do your job, and then again, you just do whatever you want. That’s wrong.” (Participant 44)
	Inclusiveness and equity: assurance that AI helps achieve inclusiveness in designing, considering health equity	“Data that they have should be gone through the process of development with it [AI], where the clinician is happy, because ultimately they will be the ones that take the camp for the diagnosis.” (Participant 20) “…If they [researchers] do not involve the community, what was the app is going to be for? Rural and remote, incidentally. So, they need to be involved, like co-design.” (Participant 47)
**Performance reliability**		
Refer to factors related to performance of health systems, with the use of AI to deliver care	Timeliness and efficiency: timely and efficient care provision	“…This lady is seriously unwell. Just get her in and give her the meds or whatever…” (Participant 27) “…Something like diabetes can fluctuate so widely within a single day. Having that instant feedback versus waiting. It’s a Friday afternoon, if something is wrong, and you can’t get easy or quick or cheap help until Monday…” (Participant 29)
	Personalization: providing care based on consumers’ concern, needs, and expectations	“This is exactly the type of product that I needed and wanted and…beneficial…it comes down to the outcome of what personalization looks like….” (Participant 18)
	Accuracy and reliability: providing correct and reliable recommendations	“AI is still unpredictable. It’s still learning…I’ve heard of different scenarios where the AI will actually come up with different scenarios that are not evidence based, because it wants to please you.” (Participant 50)
	Clinician oversight: clinicians manage and control AI, incorporate with their expertise, and make final clinical decisions	“So, it is you asking a question, and maybe we would say we used to ask another colleague, but we’re asking AI to help with that situation. No, I don’t agree with what that’s done, what they’ve said, and then go and ask another colleague.” (Participant 35) “AI will bring some new technology to make our life easier and convenient, but it’s still new…We can develop and depend on the AI, but people should control the AI…” (Participant 57)

Abbreviations: AI, artificial intelligence; GP, general practitioner.

**Table 4.** Evidence-Based Recommendations for Achieving Social License for AI in Health Care^a^

Recommendations	Relevant actors	Linked themes	Quotations (participant identifier)
**Building digital foundations**			
Integrate AI into clinical workflows in ways that preserve and support meaningful patient-clinician interactions	AI developers, health service organizations, and clinicians	Relational engagement	“We’re going to end up completely removing…the person, the human practitioner…it’s frightening.” (Participant 45) “High respect professions. I will not be too worried. There’re the people actually saved my life. They know what they’re doing…” (Participant 63)
Design and implement AI as a supportive tool of clinicians rather than a replacement	Health service organizations, AI developers, and clinicians	Performance reliability	“We used to ask another colleague, but we’re asking AI to help with that situation…” (Participant 35) “We can develop and depend on the AI, but people should control the AI…” (Participant 57)
Identify effective strategies for communicating AI in health care to consumers	Policymakers, health service organizations, clinical professional bodies, and educational sectors	Structural support	“…We’re always on about how researchers or clinicians need to be trained in how to communicate with consumers...And there isn’t enough of it yet.” (Participant 34)
**Transforming care for groups of patients**			
Provide plain-language information about use of patient health data for AI, and the use of AI in patient care	Health service organizations, AI developers, policymakers, and consumers	Structural support	“I would like to know some principle they use the data,…how do they gathering the data? How do they store it, and what’s the principle they gathering the data?” (Participant 96)
Establish clear, rigorous governance standards for health care AI	Policymakers, regulators, health service organizations, clinicians, and consumers	Structural support	“…We should have the legislation actually to identify where responsibility lies.” (Participant 44)
Include affected communities in AI design and evaluation, particularly groups whose care may be shaped by the tool	Policymakers, AI developers, health service organizations, consumers, and clinicians	Structural support	“…If they [researchers] do not involve the community, what was the app is going to be for? Rural and remote, incidentally…” (Participant 47)
**Reimagining care models**			
Embed codesign with consumers and clinicians across the AI lifecycle	Policymakers, AI developers, health service organizations, clinicians, and consumers	Structural support	“Data that they have should be gone through the process of development with it [AI], where the clinician is happy, because ultimately they will be the ones that take the camp for the diagnosis.” (Participant 20) “…They [community] need to be involved, like codesign.” (Participant 47)
Ensure AI provides timely, relevant, personalized, and accurate recommendations, under active clinician oversight	Policymakers, regulators, clinical professional bodies, AI developers, health service organizations, clinicians, and consumers	Performance reliability	“This is exactly the type of product that I needed and wanted and…beneficial…it comes down to the outcome of what personalization looks like….” (Participant 18)
In short-term care settings, prioritize AI functions that provide timely, reliable support for clinician-led decisions	Policymakers, clinical professional bodies, health service organizations, AI developers, and clinicians	All themes	“…This lady is seriously unwell. Just get her in and give her the meds or the or whatever…” (Participant 27)
In long-term care settings, prioritize robust structure and patient-clinician relationships	Policymakers, clinical professional bodies, health service organizations, AI developers, clinicians, and consumers	All themes	“I would like to know some principle they use the data,…how do they gathering the data? How do they store it, and what’s the principle they gathering the data?” (Participant 96) “…If the doctor gives me like the diagnosis based on like these parameters [of AI], I would just listen to it.” (Participant 66)

Abbreviation: AI, artificial intelligence.

^a^ Recommendations were derived from the thematic analysis and are mapped to the themes and illustrative quotations presented in Table 3. The salience of each recommendation varies by care contexts. In long-term care, relational engagement and structural support should be emphasized. In short-term care, performance reliability is more salient.

### Relational Engagement

Participants expressed high trust in clinicians, viewing them as central to safe and effective care. Participants consistently emphasized that clinician-patient interaction is critical and irreplaceable. This is more salient in long-term care settings. Participants valued the continuity and the relational engagement that these long-term interactions provide.

Participants’ trust in clinicians and their ongoing relationships with clinicians influenced their trust in AI. Most preferred that clinicians inform them when AI is used and their data are used. However, recognizing clinicians’ time constraints, some participants reported they would not mind if someone else introduced this information, allowing clinicians to focus more on their care.

Participants expressed diverse views on shared decision-making about using data for AI and AI integration in health care. Some believed that explicit consent should be obtained before their health data were used for AI training. Others noted that their data had already been applied for different purposes without prior consent, and they were also comfortable with the data being used for AI. However, most emphasized the importance of being informed before health systems used health data for AI training and expected transparency when AI is implemented in their care.

### Structural Support

Participants raised concerns regarding both the use of health data by AI systems and the broader management of AI. They expressed strong expectations for the establishment of clear mechanisms to govern AI systems. Rather than positioning governance as a means to constrain AI use, participants saw governance as a necessary condition to enable and enhance its social license. This was present in all scenarios but more pronounced in long-term care scenarios. In short-term care scenarios, periods participants described as highly stressful and frightening when their lives felt at risk, these structural factors were not their priorities.

Many participants expressed their concern about data privacy, including risks of being identified, tracked, scammed, targeted with advertising, or having their data being used for commercial gain. Although some acknowledged that commercialization and the involvement of private companies could contribute to the sustainable development of AI, others expressed heightened concern about data privacy. Despite these differences, there was broad agreement on the necessity of robust mechanisms to safeguard health data and protect individual identities.

Participants underscored the importance of accountability, emphasizing both consumers and clinicians need to understand how AI algorithms operate. Although participants expressed different views on who should communicate with patients, through which channels, when, and how often, participants expressed a strong desire for transparency regarding AI in health care. They desired information on what is AI, how it is trained, how it functions and is implemented, data governance, privacy and security, and the identity of those responsible for developing and managing.

Participants expressed the importance of involving both clinicians and consumers in the development and implementation stages of AI in health care. Moreover, AI should be trained on comprehensive datasets to enable the provision of personalized recommendations.

### Performance Reliability

Participants emphasized the need for AI-supported systems to provide timely and effective feedback based on consumer data, helping save more time for patients and clinicians. This was more obvious in short-term care scenarios. Many participants emphasized that in short-term care scenarios, their attention shifted to their well-being, and they wanted to prioritize fast and efficient interventions over other considerations.

Participants also highlighted the importance of personalized care, which can support them with treatment plans tailored to their needs, concerns, and expectations. Participants expected AI-generated decisions to be accurate and reliable. Although many expressed high trust in the accuracy of AI, others raised concerns based on their personal experiences, where AI provided inaccurate recommendations.

Participants highlighted that AI should function as a supportive tool of clinicians rather than a replacement. AI can help them gather information and contribute to clinical judgment, akin to the role of another colleague. However, they stressed that clinicians must retain responsibility of managing and controlling AI, incorporating its outputs with their professional expertise, and ultimately making the final clinical decisions.

## Discussion

This study provides, to our knowledge, the first scenario-based, participatory empirical exploration of how perceived trust and benefit condition social license for AI in health care. Although prior work recognized the dynamic nature of social license,^12,21^ our study extends this literature by explicitly foregrounding its multidimensionality within social license. We identify 3 interrelated components that shape social license: relational engagement, structural support, and performance reliability, with their relative importance shifting across short-term and long-term care settings. In the settings of increasing need of refining and coordination of governance and regulatory frameworks at local, state, and national levels, our findings provide actionable guidance for the sustainable integration of AI in health care while upholding principles of patient-centered care. At the international level, these insights also align with broader efforts to strengthen AI governance globally.

Although this study’s findings align with previous studies that report generally positive consumer attitudes toward sharing data for AI and AI use in health care,^19,29,30,31^ this study goes further by reflecting social license as a whole and moves the discussion beyond a dichotomous yes or no answer toward a more nuanced perspective of “yes, if… and when…” Social license is conditional: participants are inclined to support AI if it demonstrably improved health outcomes for consumers, enhanced support for clinicians, and minimized risk.

Although the importance of data governance, safety, transparency, accountability, inclusiveness, or equity have been addressed and explored in existing AI ethics and governance frameworks^1^ and literature^31,32,33,34^ as technical or regulatory requirements, this study enhances those discussions by emphasizing that structural factors are not only for technical reliability and legitimacy but also for confidence among consumers. Future AI implementation should embed codesign with consumers and clinicians to ensure AI systems reflect their contexts and needs. We also highlight the importance of communicating these structural safeguards, underlying processes, and logics in ways that consumers and clinicians can understand and feel comfortable with, an aspect largely overlooked in previous frameworks. Further research is needed to explore effective strategies for communicating technical components to consumers.

We propose relational engagement as one of the main components of social license for AI. Although there is substantial evidence of patient trust in clinicians and importance of human interactions in AI-supported care,^35,36^ our findings extend this by examining how AI should be integrated within the patient-clinician relationship. Consumers consistently expressed that AI should enhance rather than erode patient-clinician interactions, shared decision-making, and empathy. These are vital components of health care delivery that AI cannot replace or take over. This is particularly important in resource-constrained health systems, where clinicians are overworked and have limited time to build rapport or demonstrate empathy. AI implementation should be a supportive tool to reduce administrative work and minimize time spent on paperwork and screens, enabling more meaningful patient interactions. To achieve this, AI tools should be integrated seamlessly into routine care without compromising patient-clinician interactions.

Although prior literature has emphasized that accuracy and efficiency are essential components of AI systems, these discussions are mainly grounded in technical viewpoints rather than consumer perspectives.^37,38^ Our study extends these findings by revealing that patients expect AI-supported care to deliver timeliness, personalization, and active clinician engagement. Participants emphasized that health systems should leverage AI to gather, summarize, and visualize data or generate recommendations that support clinicians while maintaining the clinician as the key care provider. This perspective reframes performance from a purely technical metric to an experiential dimension of care, highlighting the clinician’s role in sustaining trust and patient-centeredness.

We emphasize that the relative importance of these 3 components shifts across long-term and short-term care settings. We designed scenario 1 (1a and 1b) to present long-term care settings and scenario 2 (2a and 2b) to present short-term care settings. In both scenarios, the b versions introduced more ambiguity and complexity regarding transparency, data use, and organizational involvement. As anticipated, trust and perceived benefit declined from scenario 1a to 1b. In contrast, from scenario 2a to 2b, trust and perceived benefit increased. One explanation is that in short-term care situations, consumers may prioritize rapid, efficient, and life-saving decisions over data or relationship concerns, accepting more risks if AI can help achieve better outcomes. In long-term care, where urgency is lower and patient-clinician relationships develop over time, patients expect AI-supported care to emphasize relational and structural components. Recognizing the distinction between long-term and short-term care settings is essential because context-specific approaches can support achieve social license with efficient resource allocation. Future implementation of AI in health care should be guided by the demands of each care context.

### Limitations

This study has some limitations. Due to resource constraints, we included only participants from one state of Australia, who could communicate in English. Despite efforts to recruit diverse participants and provide accessibility accommodations, these boundaries may still limit the transferability to more diverse or international contexts. The scenarios were hypothetical, so practical studies are needed to validate these findings. Because our study primarily emphasized consumer perspectives, future research should integrate other stakeholders, such as developers, policymakers, and health care professionals.

## Conclusions

In this qualitative study of consumer perspectives on AI in health care, we demonstrated the first, to our knowledge, scenario-based, participatory, empirical exploration of how perceived trust and benefit conditioned social license for AI in health care, showing that social license should be understood as a conditional construct rather than a fixed state. Structural, performance, and relational factors were interrelated components that shape social license and must be considered from the consumer’s perspective. Our study offered evidence-based recommendations for stakeholders designing and implementing AI, underscoring the need for tools that support both consumers and clinicians in delivering care that was personalized, empathetic, and responsive to patient needs.

## Notes

Supplement 1. **eFigure 1.** Comfort board **eMethods 1.** Standards for Reporting Qualitative Research (SRQR)* **eMethods 2.** Scenarios **eMethods 3.** Workshop guide **eMethods 4.** Participant demographics **eMethods 5.** Data analysis **eFigure 2.** Example of thematic maps **eFigure 3.** Three conceptual “horizons” for digital health transformation

Supplement 2.
Data Sharing Statement
