# Supplementary material for: Consumer Perspectives on Trust in and Benefits of Artificial Intelligence in Health Care
Source: JAMA Netw Open. 2026 Aug 4;9(8):e2626916. doi: 10.1001/jamanetworkopen.2026.26916 (PMC13439431; doi:10.1001/jamanetworkopen.2026.26916)
Supplement: Supplementary file 1 [file jamanetwopen-e2626916-s001.pdf]

Supplemental Online Content

Duong T, Plage S, Woods L, et al. Trust and benefit for artificial intelligence in health care and consumer perspectives. *JAMA Netw Open*. 2026;9(8):e2626916.
doi:10.1001/jamanetworkopen.2026.26916

eFigure 1. Comfort board

eMethods 1. Standards for Reporting Qualitative Research (SRQR)*

eMethods 2. Scenarios

eMethods 3. Workshop guide

eMethods 4. Participant demographics

eMethods 5. Data analysis

eFigure 2. Example of thematic maps

eFigure 3. Three conceptual "horizons" for digital health transformation

This supplemental material has been provided by the authors to give readers additional information about their work.

© 2026 Duong T et al. *JAMA Network Open*.

eFigure 1. Comfort Board

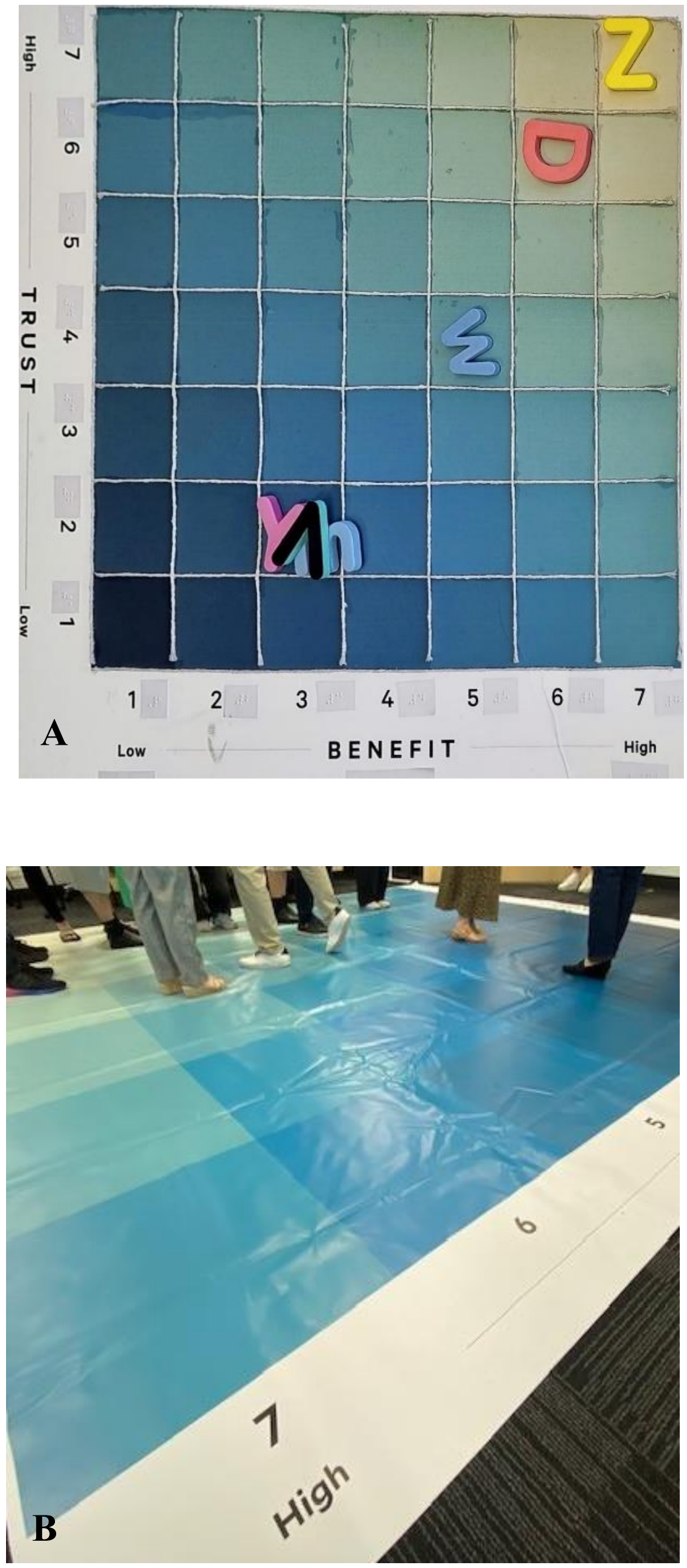

eFigure 1. Comfort Board showing participants' ratings of trust and perceived benefit

Legend: eFigure 1A illustrates the tabletop Braille version Comfort board, with participants' tokens; Figure 1B illustrates the Comfort Board with participant engagement

© 2026 Duong T et al. *JAMA Network Open*.

eMethods 1. Standards for Reporting Qualitative Research

(SRQR)*

Title and abstract

Page/line no(s).

**Title** - Concise description of the nature and topic of the study Identifying the study as qualitative or indicating the approach (e.g., ethnography, grounded theory) or data collection methods (e.g., interview, focus group) is recommended	1
**Abstract** - Summary of key elements of the study using the abstract format of the intended publication; typically includes background, purpose, methods, results, and conclusions	4

Introduction

**Problem formulation** - Description and significance of the problem/phenomenon studied; review of relevant theory and empirical work; problem statement	6
**Purpose or research question** - Purpose of the study and specific objectives or questions	7

Methods

© 2026 Duong T et al. *JAMA Network Open*.

**Qualitative approach and research paradigm** - Qualitative approach (e.g., ethnography, grounded theory, case study, phenomenology, narrative research) and guiding theory if appropriate; identifying the research paradigm (e.g., postpositivist, constructivist/ interpretivist) is also recommended; rationale**	7
**Researcher characteristics and reflexivity** - Researchers' characteristics that may influence the research, including personal attributes, qualifications/experience, relationship with participants, assumptions, and/or presuppositions; potential or actual interaction between researchers' characteristics and the research questions, approach, methods, results, and/or transferability	7
**Context** - Setting/site and salient contextual factors; rationale**	8
**Sampling strategy** - How and why research participants, documents, or events were selected; criteria for deciding when no further sampling was necessary (e.g., sampling saturation); rationale**	8
**Ethical issues pertaining to human subjects** - Documentation of approval by an appropriate ethics review board and participant consent, or explanation for lack thereof; other confidentiality and data security issues	8
**Data collection methods** - Types of data collected; details of data collection procedures including (as appropriate) start and stop dates of data collection and analysis, iterative process, triangulation of sources/methods, and modification of procedures in response to evolving study findings; rationale**	9
**Data collection instruments and technologies** - Description of instruments (e.g., interview guides, questionnaires) and devices (e.g., audio recorders) used for data collection; if/how the instrument(s) changed over the course of the study	9

© 2026 Duong T et al. *JAMA Network Open*.

**Units of study** - Number and relevant characteristics of participants, documents, or events included in the study; level of participation (could be reported in results)	10
**Data processing** - Methods for processing data prior to and during analysis, including transcription, data entry, data management and security, verification of data integrity, data coding, and anonymization/de-identification of excerpts	9
**Data analysis** - Process by which inferences, themes, etc., were identified and developed, including the researchers involved in data analysis; usually references a specific paradigm or approach; rationale**	9
**Techniques to enhance trustworthiness** - Techniques to enhance trustworthiness and credibility of data analysis (e.g., member checking, audit trail, triangulation); rationale**	10

Results/findings

**Synthesis and interpretation** - Main findings (e.g., interpretations, inferences, and themes); might include development of a theory or model, or integration with prior research or theory	10
**Links to empirical data** - Evidence (e.g., quotes, field notes, text excerpts, photographs) to substantiate analytic findings	22 (Table 3)

Discussion

**Integration with prior work, implications, transferability, and contribution(s) to the field -** Short summary of main findings; explanation of how findings and conclusions connect to, support, elaborate on, or challenge conclusions of earlier scholarship; discussion of scope of application/generalizability; identification of unique contribution(s) to scholarship in a discipline or field	13

© 2026 Duong T et al. *JAMA Network Open*.

**Limitations** - Trustworthiness and limitations of findings	16

Other

**Conflicts of interest** - Potential sources of influence or perceived influence on study conduct and conclusions; how these were managed	16
**Funding** - Sources of funding and other support; role of funders in data collection, interpretation, and reporting	17

*The authors created the SRQR by searching the literature to identify guidelines, reporting standards, and critical appraisal criteria for qualitative research; reviewing the reference lists of retrieved sources; and contacting experts to gain feedback. The SRQR aims to improve the transparency of all aspects of qualitative research by providing clear standards for reporting qualitative research.

**The rationale should briefly discuss the justification for choosing that theory, approach, method, or technique rather than other options available, the assumptions and limitations implicit in those choices, and how those choices influence study conclusions and transferability. As appropriate, the rationale for several items might be discussed together.

Reference:

O'Brien BC, Harris IB, Beckman TJ, Reed DA, Cook DA. Standards for reporting qualitative research: a synthesis of recommendations. *Academic Medicine*, Vol. 89, No. 9 / Sept 2014 DOI: 10.1097/ACM.0000000000000388

© 2026 Duong T et al. *JAMA Network Open*.

eMethods 2. Scenarios

A multidisciplinary team of clinicians, researchers, consumer representatives, and technical experts developed two realistic scenarios, which were piloted in a prototype workshop and refined before being rolled out with health consumers. These scenarios aimed to explore variations in consumer perspectives on the use of AI in healthcare, each comprising sub-scenarios a and b. The first set of scenarios focused on chronic care, while the second set of scenarios addressed acute care. Each pair of sub-scenarios differed in transparency, data use, and organisational involvement to encourage a spectrum of responses and discussions

Scenario	Summary
1a	Outpatient care settings<br>Patient was informed by a physician about the AI tool using their health data<br>AI tool selected anonymised health data
1b	Outpatient care settings<br>Patient was not informed about continuous use of their health data for AI<br>AI tool used more of their health data than 1a, including some identifiable data<br>AI tool was implemented as a health app for patient use<br>AI tool was associated with commercialisation
2a	Inpatient care settings<br>Patient was informed by a receptionist about AI tool using their health data
2b	Inpatient care settings<br>Patient was not informed about continuous use of their health data for AI<br>AI tool was implemented for clinician use<br>Private organisation was involved

Scenario 1

Imagine that you are Abi, living in suburban Queensland with your partner and 2 children. The two scenarios take you through different fictional experiences at healthcare facilities in Queensland.

1a.

© 2026 Duong T et al. *JAMA Network Open*.

You've been diagnosed with **type 2 diabetes** about three months ago. Your GP referred you to a diabetes centre at a public hospital for care. Lately, you have been going there to see your doctor regularly, who helps you manage your blood sugar levels.

To manage your diabetes, you do what your doctor says — taking three pills and two injections every day. You change your diet and exercise routines. You also wear a small device on your arm called a **continuous glucose monitor (CGM)**. This device checks your blood sugar throughout the day and sends results to your phone and your doctor. It is not easy to keep up with all these changes. Some days are harder than others. But you are doing your best, and you really hope your blood sugar will be under control soon.

Today, you go to the diabetes centre for a check-up. Your doctor tells you that the hospital is working on a new way to help with diabetes care using **artificial intelligence (AI)**. This AI is being trained using health data from all patients with diabetes in this hospital, including yours.

Your doctor explains that before the data is used in AI training, the hospital removes your name and hospital identifier. This means the data will not be connected to who you are. The AI learns from things like age, symptoms, blood test results, diagnosis, and medicines.

Your doctor says that when the AI is ready in the future, it could help doctors find early signs of diabetes problems. It might also give advice on your care plan, especially your diet, exercise and other behaviours to help prevent these problems. These can make it easier to manage diabetes, based on what each person needs.

1b.

One year later, you return to the hospital for another check-up. A nurse tells you about a **new mobile app that can help you track and manage your diabetes**.

You try the app and find it easy to use. This app quickly becomes part of your daily routine. The app feels like a virtual assistant. It gives you advice that fits your lifestyle and helps you make choices about food, exercise, and medicine. You feel like it understands your needs and gives helpful tips at the right time.

One day, while looking through the app's settings, you tap on 'About this app'. You find out that **the app runs on the same AI tool your doctor mentioned last year**. This AI is **still**

© 2026 Duong T et al. *JAMA Network Open*.

learning from health data from all patients with diabetes in the hospital, including yours.

As you read more, you find out that the AI tool uses many types of information. This includes age, symptoms, test results, diagnosis and medicines. **It also uses your daily blood sugar readings from the CGM, your diet and exercise, your social background, family history, and information from your mobile health apps.**

At the bottom of the page, you notice a message: **"This app is free for the first two months. After that, it costs $1 per month."**

Scenario 2

Imagine that you are Toni, living in rural Queensland with your partner. The two scenarios take you through different fictional experiences at healthcare facilities in Queensland.

2a.

A few days ago, you felt a bit tired and had a small cough. You thought it was just a cold, so you stayed home to rest. But tonight, while eating dinner with your partner, **you suddenly feel very sick. Your chest hurts, you have a high fever, and your cough gets much worse. You feel weak and scared**.

Your partner takes you to the hospital right away. The doctors say you have a serious lung infection and need urgent treatment. You are admitted to the hospital for care.

While you are being admitted, a hospital staff member at the front desk tells you that the hospital is working on a new way to improve care using **artificial intelligence (AI)**. This AI is being trained using health data from all patients in this hospital, including yours.

The hospital staff member explains **that before the data is used in AI training, the hospital removes your name and hospital identifier. This means the data will not be connected to who you are.**

The staff member says when this AI tool is ready to use in the future, it may benefit you and other patients in the hospital. It could help doctors act faster when patients get worse. It might also give advice to help treat each patient safely and quickly, based on each person situation and needs.

© 2026 Duong T et al. *JAMA Network Open*.

2b.

Two years later, you return to the same hospital. This time, you are in **severe pain** — your right leg **hurts more than anything you've ever felt before**. You can't walk and need help right away. You are admitted to the hospital for care.

During your stay, **you note the healthcare team acts fast, your test results come back quickly, and your treatment plan is made and put into practice quickly.** You start to feel better soon.

A few days later, as you are recovering in hospital you browse the internet to pass the time. **You find out that the hospital is using the same AI tool the staff member mentioned two years ago to help with patient care**. **It occurs to you that the care you received a few days earlier, was based on a treatment plan produced by AI.**

As you read more, you learn that the hospital is now working with **a private company to make the AI tool better.** This will help the AI learn faster and give better help to doctors. It may also help hospitals save money when they use AI for a long time.

© 2026 Duong T et al. *JAMA Network Open*.

eMethods 3. Workshop guide

Engaging consumers to support community approval and ethical implementation of artificial intelligence in clinical practice

Please note: This guide indicates how we aim to structure the interactive workshops. We will aim to be guided by participants in their willingness to disclose and reflect on their own levels of comfort using this participatory methodology. The workshop will be divided into the following three parts:

Part 1: Getting to know each other, building rapport and easing into the topic

1. Introductions: Tell us briefly about yourself. What brought you here today? What perspectives and experiences do you bring to our discussions on using health data to train AI models?
2. Brief introduction to project, the emergence of AI in healthcare, privacy, workshop structure and house rules

AI Introduction:

So, what is AI?

AI stands for *artificial intelligence*. It's a kind of smart technology that helps computers and devices do things that usually need human thinking — like learning, solving problems, making decisions, or understanding information.

How is AI created?

To create AI, people write a set of instructions called an *algorithm*. These instructions tell the computer what to do with data. Then, the AI is trained by giving it large amounts of data and uses it to improve its performance. Over time, it learns patterns and gets better at doing its job. Once it's trained, the AI can make decisions or do tasks on its own.

What can AI do in healthcare?

AI has potential in many aspects of clinical care. It's being integrated into platforms like clinical software, websites, wearable devices, smart devices (Ex smart phone) and diagnostic equipment. AI can help patients and healthcare staff with:

- Doing admin work
- Interpreting test results
- Making decisions about diagnosis and treatment

© 2026 Duong T et al. *JAMA Network Open*.

- Summarizing medical information and writing reports
- Answering patient messages

House rules:

There are a few things we'd like you to know about today's workshop – particularly given the sensitive nature of the topic.

- You will only be asked for your opinions on fictional scenarios. You're not going to be asked to share any personal details, except those that you're happy to talk about
- Nothing that is said here today will be linked to you personally in any way.
- Another way of saying this is that all opinions will be reported anonymously – comments won't be linked to you in any way after the workshop.
- We ask that any opinions that are shared today stay in this room when we all leave, to respect individuals privacy please can we request that you don't use your phones during the workshop.
- We'll be taking photos of counters on the trust/benefit grid and your feet on the mat so that afterwards we can compare positions across different workshops. We'll tell you when we're taking these photos, and no faces will be included in the photos.

3. Brief discussion on what trust and benefit mean.

Part 2: Comfort Board familiarisation - Comfort Board grid mat

4. Read out Scenario 1a;

   Encourage participants to reflect on trust and perceived benefit from the scenario.
   Ask participants to position themselves on the comfort board matrix.
   Record positions and ask participants to elaborate on their decision to move or stay put

5. Read out Scenario 1b;

   Encourage participants to reflect on trust and perceived benefit from the scenario.
   Ask participants to position themselves on the comfort board matrix.
   Record positions and ask participants to elaborate on their decision to move or stay put

Part 3: Small group discussions – Tabletop Comfort Board

© 2026 Duong T et al. *JAMA Network Open*.

6. 1. Advise participants to roundtable discussions with tabletop Comfort boards

   2. Read out Scenario 1a;

   3. Ask participants to position themselves on tabletop Comfort boards.

   4. Record positions and ask participants to elaborate on their decision.

7. 1. Read out Scenario 1b;

   2. Ask participants to position themselves on tabletop Comfort boards.

   3. Record positions and ask participants to elaborate on their decision.

8. 1. Read out Scenario 2a;

   2. Ask participants to position themselves on tabletop Comfort boards.

   3. Record positions and ask participants to elaborate on their decision.

9. 1. Read out Scenario 2b;

   2. Ask participants to position themselves on tabletop Comfort boards.

   3. Record positions and ask participants to elaborate on their decision.

Part 4: Single Question – Roundtable discussion

10. What would increase your comfort with AI in healthcare?

Conclusion:

Thank participants and remind them of opportunities to learn about the study's findings.

© 2026 Duong T et al. *JAMA Network Open*.

eMethods 4. Participant Demographics

Research Title: *Engaging consumers to support community approval and ethical implementation of artificial intelligence in clinical practice*

Date: ………………………….…….……**Location:** ……………………………………………….

Participant name (Optional):

…………………….……………………………………………………………………….

Please tell us a little bit more about yourself. This will help us to better understand how different people may have different views on AI in healthcare.

Your completion of this form is voluntary. You can skip questions you don't want to answer. You can still participate in the workshop if you don't want to share this information.

1. Which type(s) of healthcare services have you used in Queensland in the past 24 months? (Select all that apply):

☐ Outpatient care (you received treatment or consultation but did not stay overnight)

☐ Inpatient care (you were admitted to a hospital and stayed overnight or longer)

☐ I'd rather not say

2. Which Queensland Healthcare facility have you mostly used in the past 24 months?

…………………….…………………………………………………………………………

……………………………………………………………………………………………….

…………………………………………………………………………………

3. In your own assessment how much do you know about AI in healthcare? (Select one):

☐ Not familiar at all – I know very little about AI

☐ Slightly familiar – I've heard about AI but don't fully understand it

☐ Moderately familiar - I understand basic concepts and applications

© 2026 Duong T et al. *JAMA Network Open*.

☐ Very familiar - I understand how AI works

☐ I'd rather not say

4. What is your age?

☐ 18–30

☐ 31–60

☐ 61 or over

5. What is your gender?

☐ Woman

☐ Man

☐ Non-binary

☐ I use a different term

(if you wish, please specify): …………………………………………………………………..

☐ I'd rather not say

6. Were you born overseas? If your answer is 'Yes' please tell us where:

………………………………………………………………………………………………

………………………………………………………………………………………………

……………………………………………………………………………………

7. Do you identify as being a person with a disability or impairment? If your answer is 'Yes' please tell us in what way:

………………………………………………………………………………………………

………………………………………………………………………………………………

……………………………………………………………………………………

THANK YOU!

© 2026 Duong T et al. *JAMA Network Open*.

eMethods 5. Data analysis

All quantitative data and analyses were used in a supportive capacity to contextualise qualitative findings. Aggregated comfort board positions provided a visual summary of participants' comfort levels and their shifts across scenarios. Descriptive statistics were used to summarize positions distributions and changes in position means. The Wilcoxon signed-rank test was used in an exploratory manner to examine the trust and benefit changes within scenarios ($p < .05$). Analyses were conducted in R 4.4.0 and Studio R, with Python 3.11 used for visualisation.

Group discussions were audio-recorded and transcribed verbatim by TD and MD with support from Otter.ai. Transcripts were analysed using abductive reflexive thematic analysis approach in NVivo 14. Themes were developed iteratively, guided by a conceptual breakdown of social licence, including trust and perceived benefit. Additional constructs were generated inductively from the transcripts. TD, SP and MD independently coded the first transcripts to generate an initial coding framework, which was then refined through regular meetings where discrepancies, emerging patterns, and alternative interpretations were discussed. As analysis progressed, the coding framework was revised, updated new insights and merging overlapping codes. Broader team meetings were used to finalise themes. Participant-generated thematic maps were compared with the researcher-generated coding framework, and insights from this comparison informed the refinement of codes and the re-organisation of themes. An audit trail documented key decisions throughout the study. Insights from the thematic analysis were synthesised into evidence-based recommendations for achieving social licence and mapped to relevant actors across three conceptual "horizons" of digital health transformation framework (eFigure 2)

© 2026 Duong T et al. *JAMA Network Open*.

eFigure 2. Example of thematic maps

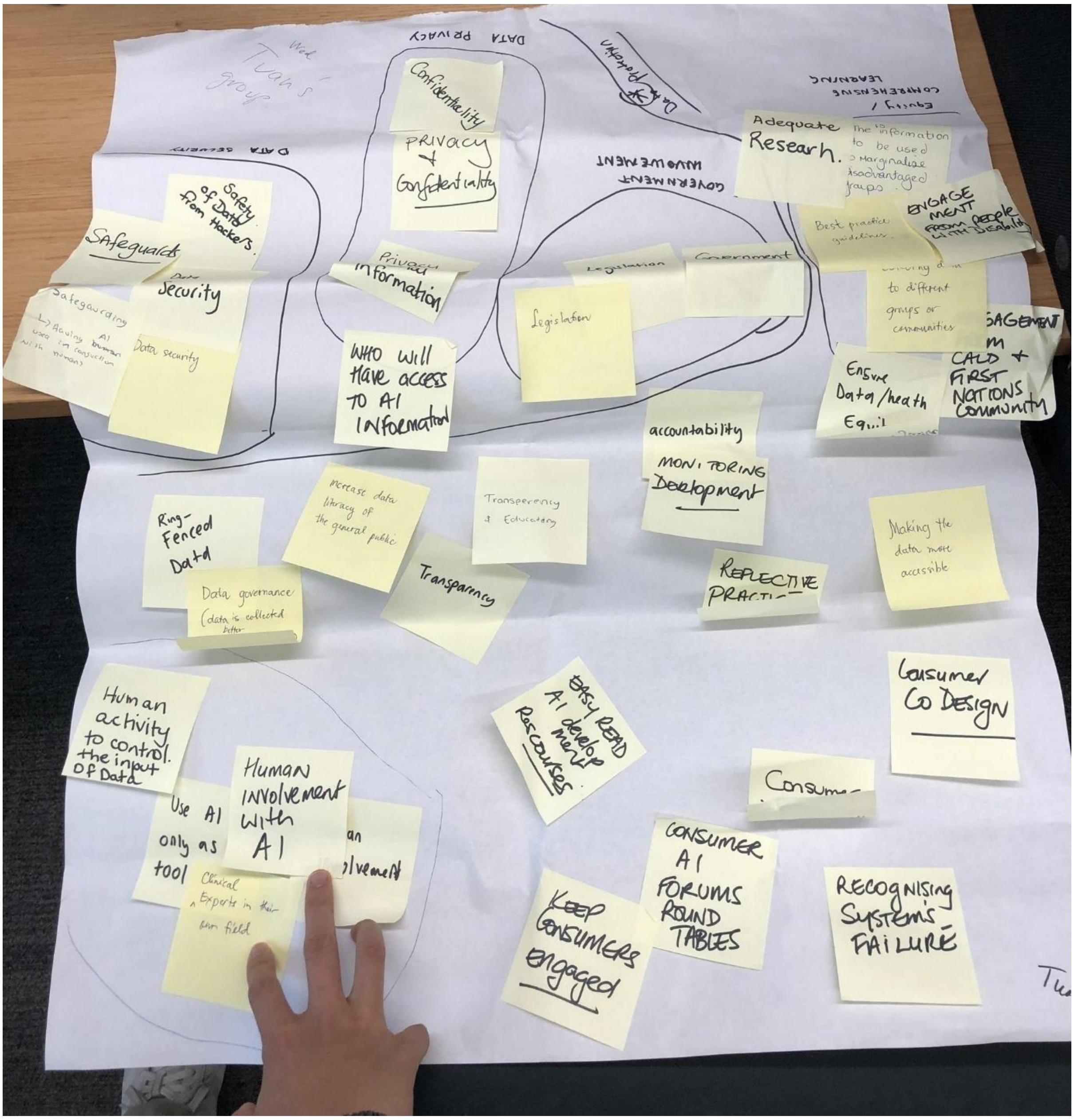

© 2026 Duong T et al. *JAMA Network Open*.

eFigure 3. Three conceptual "horizons" for digital health transformation

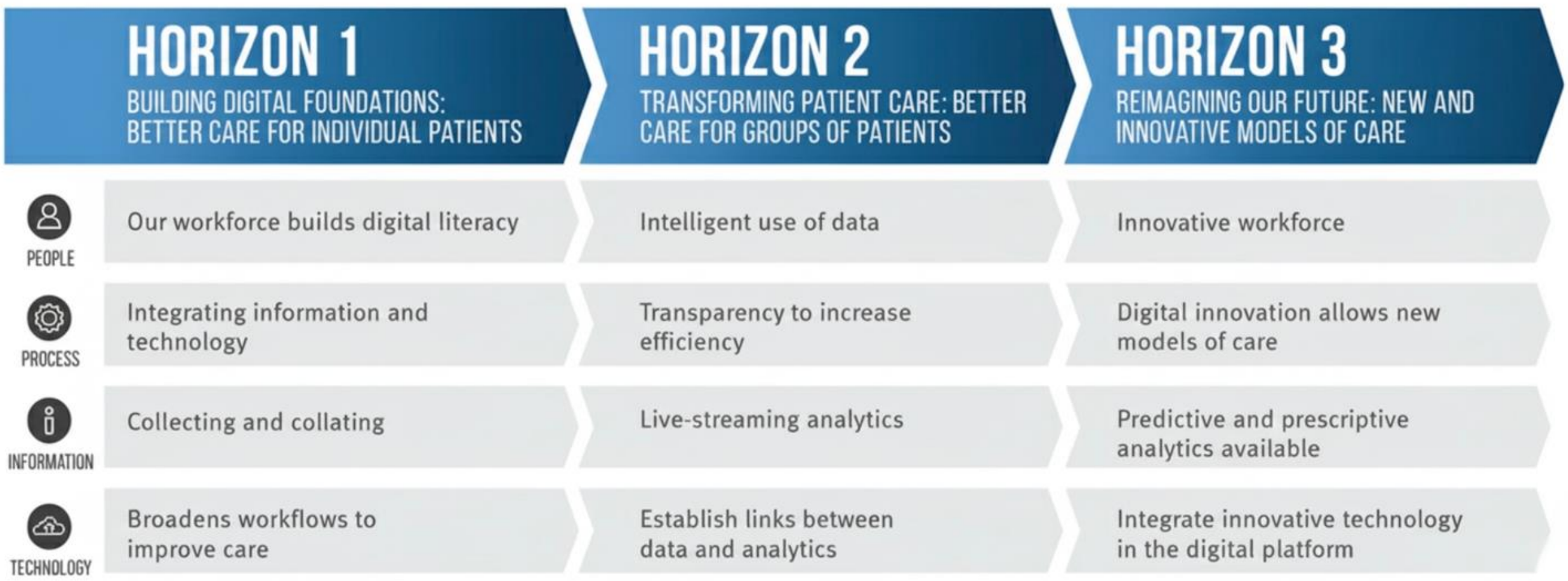

Figure 2. Three conceptual "horizons" for digital health transformation[1]

Reference

1. Sullivan C, Staib A, McNeil K, Rosengren D, Johnson I. Queensland Digital Health Clinical Charter: a clinical consensus statement on priorities for digital health in hospitals. *Aust Health Rev*. Sep 2020;44(5):661-665. doi:10.1071/ah19067

© 2026 Duong T et al. *JAMA Network Open*.
