# Supplementary material for: Consumer Perspectives on Trust in and Benefits of Artificial Intelligence in Health Care
Source: JAMA Netw Open. 2026 Aug 4;9(8):e2626916. doi: 10.1001/jamanetworkopen.2026.26916 (PMC13439431; doi:10.1001/jamanetworkopen.2026.26916)
Supplement: Supplementary file 2 [file jamanetwopen-e2626916-s002.pdf]

Data Sharing Statement

Duong. Consumer Perspectives on Trust in and Benefits of Artificial Intelligence in Health Care. *JAMA Netw Open*. Published August 04, 2026.
doi:10.1001/jamanetworkopen.2026.26916

Data
Data available: No

Additional Information
Explanation for why data not available: The dataset contains individual-level responses that may carry a risk of re-identification
